# Engendering Harm: A Critique of Sex Selection For “Family Balancing”

**DOI:** 10.1007/s11673-017-9835-4

**Published:** 2018-01-24

**Authors:** Arianne Shahvisi

**Affiliations:** Brighton & Sussex Medical School, University of Sussex, Medical School, Biology Road, Falmer, BN1 9PX UK

**Keywords:** Sex selection, Sexism, Assisted reproductive technologies, medicalization

## Abstract

The most benign rationale for sex selection is deemed to be “family balancing.” On this view, provided the sex distribution of an existing offspring group is “unbalanced,” one may legitimately use reproductive technologies to select the sex of the next child. I present four novel concerns with granting “family balancing” as a justification for sex selection: (a) families or family subsets should not be subject to medicalization; (b) sex selection for “family balancing” entrenches heteronormativity, inflicting harm in at least three specific ways; (c) the logic of affirmative action is appropriated; (d) the moral mandate of reproductive autonomy is misused. I conclude that the harms caused by family balancing are sufficiently substantive to override any claim arising from a supposed right to sex selection as an instantiation of procreative autonomy.

## Introduction

At the start of 2015, a Bill was debated in U.K. parliament which attempted to criminalize sex-selective abortion. While the Bill was ultimately rejected (Department of Health 2015), its driving concerns are worthy of further discussion. Though conventionally associated with particular cultures and locations (e.g. India, China, and South Korea), mild son-preference is in fact observed across many cultures globally,^1^ especially for first-borns (c.f. Bandyopadhyay 2014; Steinbacher and Ericsson 1994; Pebley and Westoff 1982), and while sex selection may not be common across all geographies, sex differential treatment of offspring is widely observed (Van Balen and Inhorn 2003; Hill and Upchurch 1995). Given that subtle and overt male privilege is written into social structures globally, son-preference may be seen as a rational choice,^2^ at least in the short term.

Most analyses voice reservations about unregulated sex selection, firstly because of consequentialist concerns about the unpredictable effects of upsetting the sex-ratio in the population at large, second because the very act of sex selection is sexist. While many commentators express moral condemnation (Seavilleklein and Mudde 2010; Strange and Chadwick 2009; Seavilleklein and Sherwin 2007; McDougall 2005), and recommend either prohibition or strict regulation, others, whose work centres on procreative liberty (e.g. Savulescu 1999; Robertson 2001; Harris 2005; Dahl 2004), argue in favour of sex-selection as an exercise of reproductive autonomy.

Amongst those opposed to non-medical sex selection, an exception is often made. Sex selection for the purposes of “family balancing” is generally considered to be permissible, often with little or no explanation. Dickens (2002, 336) considers that “prohibitions are unnecessary and oppressive where there is no sex bias but only a wish to balance a family with children of both sexes.” Grant (2006, 1659) remarks, without further comment, that “there appears to be some consensus that a sex preference is ethical if the existing family is a single sex-sibship.” McMillan (2002, 30) supports sex selection for “family balancing,” and recommends that potential abuses be minimized by changing the U.K.’s Human Fertilisation and Embryology Authority guidelines to match those of U.S. private clinic Microsort (2014), who perform non-medical selection only when “the applicants are married, have at least one child, and want to select for the sex that is less common in the family.”^3^ Pennings (1996, 2339) notes that “family balancing is intuitively accepted by a majority of the population […] In Western societies, parents wish to have a well-balanced family in terms of sex.”

Libertarian ethicists argue that one should not restrict freedoms where one cannot demonstrate harms; therefore, they believe that sex selection for the purposes of “family balancing” should be permitted. Provided it is adequately regulated, it is argued that sex-selection amongst sibling groups for the purposes of “family balancing” will not upset the population sex ratio. Further, proponents claim that sex selection for “family balancing” is not motivated by, and does not motivate, sexism, since it is chosen in response to the sex(es) of existing offspring. I will show that this second justification is mistaken and will present additional reasons for maintaining a moral condemnation of sex selection, even in cases of “family balancing.”

I will not address the claim that sex selection for “family balancing” is preferable to sex selection *simpliciter* because it does not upset the population sex-ratio but will say only this: the surest way to not upset the population sex-ratio is to permit no sex selection for any reason. Biology ensures parity (to all intents and purposes) in the adult sex ratio (Hesketh and Xing 2006). “Fixing” local, entirely random, variations in sex-ratios may affect the overall ratio in some way, because there will likely be trends in the views of the parents who are perturbed by family “imbalance” which may amount, say, to a net son-preference (this trend is in fact noted in empirical work by McGowan and Sharp (2013, 284)). Regardless, it is not clear that a slightly “upset” population sex-ratio would in itself be a problem, even if it may be a *symptom* of a very serious social issue. The reasons for concern offered in the literature are themselves far more concerning. For example, Dahl suggests that an uneven sex ratio would lead to “an overabundance of males who, once grown up, will be unable to find a mate and may resort to prostitution, molestation, rape, and other sex related crimes” (2005, 88). It seems odd and irresponsible to suggest that these crimes can be prevented by the presence of greater numbers of women, rather than changes in underlying cultures which underwrite violence against women and girls.

My discussion will close with the conclusion that sex-selection for the purpose of “family balancing” is indefensible and the recommendation that it should be legislated against. In most jurisdictions—including the United Kingdom—this is already the case, but the prohibition may founder as genetic selection is normalized for other purposes. As with any prohibition on moral grounds, convention dictates that one must swim upstream against the presumed primacy of individual liberty (c.f. Dahl 2004). I have my own views on the ideology of this presumption but will dutifully take on the challenge here. My aim is therefore to show that the harms incurred if sex-selection for “family balancing” is permitted are sufficiently great to outweigh claims of the right to individual reproductive autonomy. I will argue that the concept of “family balancing” is harmful because it:

1. medicalizes the *family*;
2. is heteronormative (i.e. sexist, homophobic, and transphobic);
3. appropriates the logic of affirmative action;
4. is an abuse of reproductive autonomy.

One might wonder why such a specific theme within the topic of sex-selection is worthy of lengthy analysis. Family balancing is a common motivation for sex selection in Western contexts, with studies showing that 30 per cent of those surveyed in Germany and 68 per cent of those surveyed in the United Kingdom (Dahl et al. 2003) desire an equal number of sons and daughters. Since the literature frames “family balancing” as the most legitimate purpose of sex selection, if this rationale can be shown to be indefensible, or shown to be no more defensible than justifications which have been dismissed on other grounds, then the overall case in favour of sex selection is weakened.

The text is organized as follows: Section 2 introduces sex selection and “family balancing.” Section 3 describes the way in which the rhetoric of “family balancing” medicalizes the family unit. Section 4 explores the various problematic ways in which sex selection invokes heteronormativity. Sections 5 and 6, respectively, propose that “family balancing” illegitimately co-opts the logic of affirmative action and is a misuse of the broader principle of reproductive autonomy. Section 7 concludes.

## What is Family “Balancing”?

First, a note on language. I adopt the terminology most commonly used in this literature and refer to “sex selection.” This is apt in one sense, since it is the sex of a future child that is being selected using available technologies. It is not so apt in another sense, since the intention of the parent is invariably to produce particular stereotypical gendered features; that is, parents wish to raise a “boy” or a “girl” according to the social meanings of those terms (Nugent 2013; Kane 2009; Goldberg 2009). I will describe the sex-gender distinction in more detail in section 4 but suffice to say that my writing will intentionally move between the terms sex (selection) and gender (selection) in order to track the correct referent for that particular context. If this seems confusing, it is because the terms are being conflated by those requesting sex selection.

Sex selection may be achieved in any of four ways. In increasing order of moral concern: (a) pre-conception sperm sorting may be used to choose an X or Y chromosome, (b) pre-implantation embryo sorting may be used to select an embryo of the correct sex, (c) an abortion may be carried out following the determination of the sex of the foetus, or (d) the child may be killed, malnourished, or refused medical attention after birth. Only (c) is currently legal in the United Kingdom, but all are in operation globally.

Left to the vagaries of nature, the population sex-ratio tends to be around 105–107 male births for every 100 female births (Hesketh and Xing 2006). Since the egg’s surface is as receptive to X-chromosome sperm cells as to Y-chromosome sperm cells, the selection of chromosomes is essentially random, with the minor departure from parity believed to be due to an evolutionary selection effect, since males have higher mortalities at all ages. This approximate parity means that families which end up being “imbalanced” are completely *normal*, in the statistical sense, as are families which end up being “balanced.” Since parents on average produce two to three offspring, the probability of having children with the same chromosomal sex is between 25–50 per cent, which is within the realm of the frequently occurring. Those who feel “cheated by nature” or that they are “playing with loaded dice” because of family “imbalance” (Pennings 1996, 2340) are misunderstanding how probability works.

“Family balancing,” or “gender variety,” is sought when a family is deemed to be “imbalanced,” that is, when the “variety” of gender amongst the sibling-group is not sufficiently great. An “imbalanced” family is a necessary condition for the perceived need for “family balancing.” Therefore, we first need a definition of an imbalanced family.

Of course, “family balance” and “gender variety” are subjective. Some parents will deem their offspring-group of two daughters and a son to be balanced, some will find their four sons to be a balanced set of siblings, while others would describe the same groups as imbalanced. Our definition should therefore capture the “target” concept—what ethicists and legislators would have “family balancing” mean—rather than the “operative” concept—the range of uses which parents employ in practice (c.f. Haslanger 2012). There are several possible target definitions. I propose the following be taken as the target definition of family imbalance:

A family is “imbalanced” if there are n more siblings of one sex than the other, where n>1 (which discounts the trivial imbalance of only children and odd-sized groups)

Desiring or pursuing “balance” relies on two assumptions: (a) it is favourable to have (close to) equal numbers of chromosomal/genital male and female children within a family; (b) family sex “imbalance” can be addressed only through “balanced” sibling groups, not through contact with other relatives or “balanced” communities of non-relatives. Both of these assumptions are questionable.

Consider that if “family” refers to a large set of extended relatives (whether genetically related or not) living together or spending time together regularly, it is not clear why the “balancing” could not be achieved by others within this group (cousins, say). As a target concept then, “family balancing” must be assumed to apply only to genetically-related offspring, from which we can infer that “family” in this case means the conjugate of two generations: parent(s) and genetically-related offspring.

Further, the “variety” on which “balance” is predicated relies on a well-articulated notion of similarity and why it is undesirable in this context. *Prima facie*, since similarity, including sex-similarity, is something many people seek in their social interactions (e.g. men and women have been shown to have a preference for same-sex friendships (Rose 1985)), any assertion that it is a negative property along some dimensions, for some social subsets, will require specific justification. Advocates of “family balancing” must explain why sex-similarity in a sibling group is deemed to be negative.

They might argue that sex diversity is specifically important for the development of children. Yet since parents tend not to be concerned if their children’s playmates share the same gender (or indeed, are stratified by class or race, as very often happens), the desire for variety within a sibling group warrants further scrutiny. One might argue that there is something unique about the sister–brother relationship that is being sought. Perhaps proponents of this view believe that sister–brother relationships are important precursors for heterosexual adult relationships. Even apart from its troubling heteronormativity, this view has an incestuous undertone that is morally discomfiting.

More judiciously, proponents may believe that children need to be adequately prepared for a world of more than one gender. This relies on the idea that determining the sex of a child will guarantee the required gendered differences (a point I’ll return to in more detail in section four). In any case, children freely associate with others at school from an early age, and there is no evidence to suggest that being brotherless or sisterless impairs a child’s ability to associate with others in adult life. Indeed, using data from the Office for National Statistics (2013), one can estimate that 67 per cent of children in U.K. families are brotherless or sisterless. Yet the literature contains claims such as this: “it is socially unhealthy for a child to grow up with siblings exclusively of the same sex, since present day society increasingly involves people of both genders interacting together, both at work and play” (Heng 2006, 319). One might instead argue that early instances of these increasingly common interactions will themselves suffice as preparation for future interactions in work and play, as they seem to for two-thirds of the population.

If arguments pertaining to the importance of diversity to child development are unconvincing, as I deem them to be, this supports the hypothesis that sex selection for “family balancing,” like sex selection more generally, simply serves a preference held by one or both of the parents. It is not a particular sibling relationship that is being sought but a particular parent–child relationship. In this sense, it is a little misleading to use the term “family balancing.” The child, who cannot choose her parents, has no say in the sex-character of her intergenerational relationships; the variety is chosen for the parents’ satisfaction, and “family balancing” for sex selection is properly understood as another instance of the genetic selection of children for the satisfaction of parents’ preferences.

This intuition is borne out by empirical work exploring the chief motivations for family balancing. Sharp et al. (2010), when questioning parents about their decision to avail themselves of sex selection technologies, discovered that “family balancing” was the prevailing motivation. Couples cited the desire to limit the size of their families, yet have a desirable offspring ratio. There was concern that without sex-selection, they might be compelled to have further children in order to pursue “balance” and “complete” (841) the family. McGowan and Sharp (2013, 282) confirm this socioeconomic concern about limiting family size while pursuing a particular sex-ratio. Another common motivation was the belief that the child would benefit from a female (or male) “influence” (McGowan and Sharp 2013), and that parents would enjoy varied rearing experiences. Pressures to conform to a presumed ideal were reported, with parents having strongly internalized a particular exemplar, e.g. “We love our daughter, and we always thought it would be perfect to have one of each. A balanced family” (Aghajanova and Valdes 2012, ¶4) or “society has this neat little box of a boy and a girl and that’s your perfect family” (Sharp et al. 2010, 842). In the next section I problematize the medicalization of departures from the norm of this “perfect family.”

## Medicalization

One of the most interesting aspects of the “family balancing” argument remains unexplored in this literature. What does it mean for properties of *families* to be the target of medical interventions? Medicalization constructs deviance as it simultaneously creates and enforces norms, with the credibility and authority of medicine conferring the ability to produce compelling notions of normality and abnormality.

Both “family balancing” (the U.K. term) and “gender variety” (the U.S. equivalent) are rhetorically-loaded terms: they are value-laden even without expansion or explanation. Balance and variety are manifestly favourable properties, therefore designating their referents in this way produces a positive, desirable impression before one has the details of their meaning or justification. Even prior to the assignation of values, the very act of identifying and naming a state, particularly with the legitimizing power of the medical establishment, is efficacious. Attention is drawn to that state, rendering it conspicuous, and, in this case, apt for moral judgement, while before it may not have existed in any meaningful sense. As Holm (2004, 32) points out, the term “family balancing” socially constructs the state of being in an unbalanced family. A randomly occurring, and morally neutral, actuality becomes a site for moral deliberation.

In other words, these terms socially construct *imbalanced* and *unvaried* families which may then be “treated” or “corrected” via medical technologies. Articulating offspring sex-distribution in terms of imbalance renders the situation ripe for medical intervention, where the pathologized subject is the *family*, rather than the individual. The conceptual framework of disease is transposed at the level of the family, and a medical intervention is presented in order to address this. Here, the word “imbalance” plays a key rhetorical role, since it is redolent of the fact that diseases and disorders can be described as bodily imbalances, so much so that one might even see the central task of medicine as redressing bodily imbalances. Speaking of “family imbalance” carries over this familiar medical paradigm of balancing imbalances to the family, pathologizing those with particular offspring sex distributions.

The pathology operates at two levels. First, the family is socially constructed as imbalanced by the terminology and hermeneutics of “family balancing,” then second, the parents (and, perhaps, any existing siblings) are assumed to be subject to some kind of actual or potential psychological disorder as a result of the imbalance. (As with other forms of social construction, the ensuing construction is causally efficacious.) Most “imbalanced” families will never know they are “imbalanced,” and, if told, would be indifferent to the label or would reject its ideology. So one must, in addition to being aware that something is “wrong,” be affected by that wrong, by feeling wronged. Some writers describe the stress of parents whose families are “imbalanced,” a stress so great that people may refrain from having another child in case the “imbalance” is made worse (e.g. Robertson 2001, 4).

What are the consequences of medicalizing the family in this way? First, it tends to entrench the nuclear family as a “natural,” self-evidently significant social unit and normalizes the isolation and self-sufficiency of that unit. Depending on one’s views, this may be seen as unduly traditional given the many varieties of family now in common existence and the porosity of their boundaries. More significantly, treating family units as disparate objects whose properties cannot be affected by society at large is both false and morally suspect. Our genders are formed largely in response to our societies, and very many of our needs and desires are formed and met by our societies. Our families are not the only social units that are responsible for our welfare, nor should they be.

The narrow application of “balance” must also be interrogated. “Balance” and “variety” in intimate social groups generally relate to personal properties other than chromosomal sex.^4^ There are many ways to be a “balanced” family, and variation within families is dependent on factors beyond the gender of siblings. Child development studies demonstrate that behaviours and character traits amongst siblings are a complex function of many interacting features, within which age-spacing and birth-order are at least as determinative of sibling interactions as sex (Minnett et al. 1983; Pepler et al. 1981). Children of different sexes with similar interests, personalities, and beliefs may not seem especially balanced. It is unclear to what extent sex-variety would guarantee or even increase the probability of any kind of meaningful “balance” or “variety,” and it is quite clear that variation in chromosomes and/or genitals is not alone a defensible aspiration.

If varied rearing experiences and a diverse household dynamic are the desired outcomes, then the parents would do better to control for aspects of intelligence, creativity, and sporting prowess, by selecting supposedly concomitant gene sequences. Of course, these forms of genetic selection are generally condemned, but if they were enacted solely to produce variety within a family, it would seem inconsistent to treat these choices more harshly than the desire to select the sex of a child, especially since they may be more likely to provide the variety that is desired. Alternatively, parents determined to have variety amongst their children might instead adopt differential parenting styles for each child: for example, bring strict and stipulative with one and lenient and liberal with another. Of course, moral issues abound, but it seems that worries about sex selection for family balancing differ from these forms of selection only in degree. Sorting sperm with particular presumed IQ-genes does not seem obviously different to sorting sperm with particular sex-genes. My point here is not an objection to the use of reproductive technology *per se* but rather a request that we be cognizant of the limitations of those technologies, given the strongly determinative influence of socialization on children (a point I revisit in section 4) and be mindful of the way in which the attitudes underwriting selection may undermine the widely held ideal of parenting children with open futures (Davis 2009, 1997).

It is instructive to question whether this “variety” is so important after all. There doesn’t seem to be anything concerning about a family in which the siblings are intent on emulating one another, as so often happens, with younger siblings imitating older siblings (e.g. Barr and Hayne 2003; Minnett et al. 1983). Indeed, one might argue that this could create a sense of solidarity and commonality which would strengthen the siblings’ relationship. It seems inevitable that there will be similarities within sibling groups that might not be desirable within a community as a whole. This is one reason why we (should) actively seek diversity in our communities and, if we can achieve it, there seems to be no reason to be concerned about a degree of unavoidable similarity within families.

Sex selection for reasons of family balancing is a way of using medical technologies to determine the sex distribution within a family, even though there is not sufficient concern about welfare to justify medical intervention. Using medical technologies to determine the properties of a collection of people, such as a family, for non-medical reasons, is clearly a case of medicalization. The ethics of medicalization centres on the concern that medical interventions applied in non-medical contexts and to non-medical subjects may become sufficiently common that people become dependent upon them and are less able to cope with aspects of their lives that are entirely natural (Illich 1976). In this sense, the medicalization of “family imbalance” may be seen as causing harm. Employing medical authority in the portrayal of particular families as aberrant or deficient seems intuitively dishonest and is certainly unproductive from a medical perspective in the sense of increasing, rather than decreasing, the number of pathologized subjects.

## Heteronormativity

The most obvious harm inflicted by sex selection for family balancing is the entrenchment of heteronormativity. Heteronormativity is shorthand for the conjunction of the following independent but related beliefs: (a) there are only two legitimate sexes—male and female—which are determined at conception/birth and are determinative of particular behaviours and properties which are “natural” to that sex; (b) there are only two legitimate genders—man and woman—and they match up to the two sexes in that order and are determinative of behaviours and roles; (c) either the most or the only legitimate sexual relations are between people of “opposite” sexes (and *ipso facto*, in heteronormative terms, genders) according to this schema. Heteronormativity is therefore the belief that the sex-gender-sexuality binary is real, immutable, and definitive of human beings and their relationships.

Heteronormativity is a set of harmful social norms. It imposes its harms in several ways. First, it delegitimizes people who do not conform to any of (a), (b), and (c), that is, intersex people, trans-people, gender-fluid people, non-binary people, and all people with non-normative sexualities. It does so passively through processes of ignoring or failing to recognize, but more often actively, via erasure and silencing, and employing shaming and violence as punishment for non-compliance.

Second, heteronormativity inflicts harms via (b): the association of particular roles and behaviours with particular genders. These roles and behaviours are socially constructed and therefore contingent, but they are not evenly or randomly distributed across the genders. Society is organized in a way that systematically privileges men and oppresses women, both overtly and implicitly. This structural inequality is known as “patriarchy” and is global and pervasive. Symptoms of patriarchy are evident in, for example, the global pay gap, the limited range of employment, educational, and leisure opportunities afforded to women as compared with their male counterparts, the undervaluing of female babies (consider female foeticide), the violence and control exercised over women, and the way in which women’s sexual autonomy is limited or denied. Men have more social power, which means that social possibilities which are respected and viewed positively are usually associated with men, while social possibilities that are limited, demeaning, and undesirable are associated with women. So, whilst both sides of the binary are restricted by the properties and roles that are normative for them, men’s options are more rewarding, valued, and extensive than women’s. Sexism describes the harms women experience as a result of heteronormativity and patriarchy.

In the next three subsections, I explore the ways in which heteronormativity and patriarchy collude so that sex selection, even for “family balancing,” is problematic. I will consider the way in which sex selection for “family balancing” exhibits and entrenches sexism (4.1), homophobia (4.2), and transphobia (4.3).

### Sexism

Much of the literature on sex selection betrays a striking misunderstanding of sexism and a rather surprising disregard for the large and rigorous body of feminist scholarship which provides ample choice with respect to definitions and examples of sexism. Instead, several prominent writers make the blunder which philosophy undergraduates are warned against, and use loose approximations, or worse, dictionary definitions of sexism, rather than consulting an academic text. (for example, Robertson (2001, 5) bases his understanding of sexism on the Compact Oxford English Dictionary’s definition). As a result, they end up with thin, inadequate, and outright false definitions, which may be faithful to colloquial usages of the term, but are inadequate bases for establishing philosophical arguments.

Consider the following contention, which I have distilled from its various equivalent appearances in the literature:

*Family balancing addresses “sex-imbalance” amongst a sibling group, therefore it cannot be sexist, since sexism is a preference for one sex.*

Other baffling, unjustified claims include this one (Leiter 2014, 2): “the goal is for gender variety in a family, and is frequently motivated by the female partner, and thus is not inherently sexist, as it may be motivated by the desire to rear children of both genders.” Is the author claiming that women cannot be sexist? Use of the definite article also indicates an assumption of heterosexuality. Further, the assumption seems to be that “the female partner” will be responsible for the child-rearing.

Likewise, Dickens (2002, 336) claims that sex selection is “clearly sexual, but not necessarily sexist” without exploring the possibility (or, as it will likely turn out, *impossibility*) of a non-medical preference being sexual yet not sexist.

In fact, this is not the correct definition of sexism, which is the crux of why these arguments fail. Sexism relates to the existence of rigid, unequal, and prescriptive gender roles, and the way in which privilege and oppression accrue on each side of the aforementioned “heterosexual matrix” (Butler 1990).

Consider, for example, that sexism towards women could be consistent with universal daughter preference. Parents might believe that girls are docile and naturally suited to housework and desire a daughter in order to have additional help around the home. Or they might believe that boys are more academic and likely to require educational fees, in which case a daughter would be more economical. These are sexist logics which nonetheless ground *daughter*-preference. The aim of this work is not to merely problematize son-preference but also to problematize daughter-preference, on the grounds that a sex-preference of any kind contributes to the harm caused by restrictive gender roles.

Offspring sex-preference does not obviously hurt people of any gender but gender roles unambiguously *do*. And since parents requesting sex selection for family balancing cite their desire for *sex-varied rearing experiences* (Robertson 2001; Steinbock 2002), this may be seen as an intention to reinforce, or collude with, harmful gender roles. As Mudde (2010, 563) puts it:

Preferring one sex over the other in our future children and acting on that preference forces parents to place a certain kind of value on gender—and expresses a kind of desire powerful enough to motivate them to try to ensure, with reliable methods, the gender of their child.

In this case, the justifications given in the literature rely on a binary in rearing experiences. This, in turn, relies on the heteronormative idea that biologically female children afford parents a rearing experience that one would expect from a child who is gendered as a girl. It pays to try to spell out what is meant by this. Any set of characteristics that one can think to list as being typical of a “girl” as opposed to a “female” generate immediate discomfort—they are very obviously rehearsals of stereotypes. (The same goes for male-sexed children.) That these stereotyped qualities are in fact what is desired and expected by many parents is evidenced by empirical studies which reveal them as stated justifications for sex preferences (Browne 2017a; Lowe 2015). As a Human Genetics Alert campaign document (2002, 1) asks: “In how many cases where parents are ‘desperate for a girl’ will they be hoping for a loud tomboy that grows up to be an engineer?”

The same arguments can be made in relation to the desire for children to have “varied” sibling experiences (as discussed in section two). Robertson suggests that “children learn important lessons from having siblings of the opposite gender” (2004, 271), and Pennings (1996, 2342) submits that “the presence of siblings of the other sex might promote mutual understanding among the children.” Yet that could only be the case if the parents were quite sure that the other sibling would not be able to personally acquire certain valuable knowledge or experiences on the basis of their sex, which would either reflect badly on their parenting or suggest that the knowledge in question relates only to the one substantive difference—that of genitals—whose relevance to children is minor and questionable.

There seems to be a misunderstanding in the literature about the way in which rearing experiences are framed and who determines their character. Brain plasticity is now paradigmatic within the neuroscience of development: it is understood that while certain specific (non-gendered) potentialities may be genetically conferred, much of what a child becomes is a result of their early experiences. Further, innate brain-sex differences have been debunked time and again (Vidal 2005, 2012; Caplan and Caplan 2015; Fausto-Sterling 2008, 2000; Seavilleklein and Sherwin 2007). As such, when Robertson (2001, 7) says “individuals could prefer to have a boy rather than a girl because of the relational and rearing experiences he will provide” this is an inversion of what will actually happen, which is that parental engagement will, in the first instance,^5^ determine whether the male child becomes a “boy” in the stereotypical sense, while the male child will serve as a blank slate whose individual potentialities will not yet be gendered. Or equivalently troublingly, consider Wilkinson and Garrard’s (2013, 29) contention that: “the sexes differ, and may offer different possible kinds of parent–child relationships.”

Wilkinson (2008) makes the distinction between sexism as *supremacism* and sexism as *stereotyping*, arguing that while supremacist motivations may not be present, any sex preference necessarily relies on stereotyping. Whilst this is *prima facie* a helpful distinction, it rests on something of a false dichotomy, because sex-stereotyping is *always* supremacist, since the stereotypes which delimit the lives of women are more constraining to their life options and more antithetical to the possibility of happiness, fulfilment, and self-determination. As such, stereotyping, and any medical practice which draws uncritically on the false, outdated science on which those stereotypes depend, harms women particularly. As de Melo-Martín (2013, 11) puts it: “it seems reasonable to believe that the consolidation—through the blessing of modern science and medicine—of such expectations can serve only to perpetuate this limitation of life choices and to further injustice.”

### Homophobia

For a rational agent to prefer sex variety in a sibling group, sex variety must be considered to be preferable to sex homogeneity in that context. By extension, absent arguments which make clear the distinction, sex variety in other familial subsets might also be presumed to be preferable. If one generation of the nuclear family unit is deemed to be superior when sex-varied, one might extrapolate that other generations are too. This implies that sex-varied parental subsets are also preferred; that is, heterosexual parent groups are preferable.

After all, if we allow that parents are justified in their desire for sex-varied children, shouldn’t we also allow that children would be justified in a desire to be raised by sex-varied adults, even if they cannot enact that desire? Couldn’t commentators claim that just as parents have the right to sex-varied rearing experiences, so too do children have the right to sex-varied parental role models? The case may even seem stronger that way around, since parents are more necessary and influential to children than children are to parents. Yet this conclusion is undoubtedly discriminatory and offensive to same-sex parents and to same-sex couples more generally. Of course, this argument proceeds merely via analogy, and that analogy is perhaps weakened by the fact that parents and sibling-groups are very different social units. One cannot infer in a robust sense that what is preferred in relation to a sibling group would also be preferred in relation to parents nor vice versa. Yet at the very least, arguments which pertain to the advantages of being raised in a “balanced” sibling group are strongly redolent of discourses in the public sphere which insist that child development requires parenting from a father and a mother (e.g. Crouch 2015; Squires 2014). Perhaps more persuasively, arguments which rely on the importance of sex variety (either to parents in relation to their offspring sex preferences, or to children with respect to their development) play into the idea that sex variety is an important part of family life, so much so that medical interventions may be sought to ensure that a parent of a particular sex experiences life with a child of a particular sex or a child of a particular sex experiences life with a sibling of a particular sex. This norm negatively portrays some single-parent families and all same-sex parents, as it implies that they are unable to provide their children with a valuable relationship or role model. (It pays to note that harm-based arguments which claim that children are better off if raised by different-sex parents are not borne out by the empirical data. Studies show that children of same-sex parents are not disadvantaged with respect to their peers (APA 2005; Patterson 1992)). For same-sex couples, this norm also signals that their *relationships* omit a key component of human flourishing— sex variety.

To make this point more directly: any argument that a particular social unit—whether it be a couple, a family, a sibling-group, or a friendship group—would be better for its members or for those they interact with if were sex/gender-varied is liable to be used to make more general arguments about the importance of sex/gender-variety in human relations that may be used to delegitimize non-heterosexual couples. At the very least very least we should be wary of those arguments. As McGowan and Sharp (2013, 288) note, sex selection for family balancing “demonstrates that the vision of the nuclear family of a father, mother, son, and daughter has been deeply normalized.” In short, aside from the cases of legitimate affirmative action I discuss in section 5, all claims that sex/gender-variety is preferable seem to be prejudiced against non-normative couplings. This is not to claim that parents wishing to sex select for reasons of family balancing are consciously homophobic, but that the norms that their desire invokes are worryingly similar to those that are used to justify homophobia.

### Transphobia

Proponents of “family balancing” adopt a realist attitude towards the heteronormative matrix. Specifically, since they are invariably aiming for gender properties as they select sex properties, parents are acting on the belief that “biology is destiny,” otherwise known as biological determinism. This belief is empirically unfounded (see e.g. Vidal 2005, 2012); doctors perpetuating this myth by indulging parents’ desires for particular gendered properties via the selection of chromosomes would be acting dishonestly (c.f. Browne 2017a).

Asserting, and thereby entrenching, biological determinism marginalizes trans-people, whose existence demonstrates that biological determinism is not a complete picture of how people experience their sex and gender identities. In addition, requesting a child of a particular sex/gender indicates an aversion to the child’s potential need to transition to another sex/gender. Selecting children *for* their sexed bodies or gender expression is antithetical to their potential need to transition away from that expression/body.

In a paper which explores the virtue ethics of parenting, McDougall (2005, 604) presents a thought experiment in which a bacterial infection initiates a sex inversion process in small children. She notes that “parents who rejected their daughter once she became a son, or vice versa, would act wrongly, just as parents who rejected their child once some other medical condition had radically affected his or her characteristics act similarly wrongly.”

One might reasonably argue that a parent who decides to select the sex of their child would not necessarily be unsupportive in the event of that child’s need to transition. Yet it seems likely that a person whose attitude to sex and gender is such that they believe that selecting the desired sex will ensure a particular gender, so much so that they are prepared to actively ensure that they produce a child of that sex (at some personal inconvenience and/or cost), would be unlikely to also accept the view that not only do chromosomes not guarantee a particular gender, they also cannot guarantee a particular sex. Indeed, empirical studies show that holding normative beliefs about gender, gender roles, and the sex-gender binary (such as those held by parents who believe that sex reliably delivers gender traits) is a strong predictor of negative attitudes towards trans-people (Norton and Herek 2013; Riggs et al. 2012; Costa and Davies 2012). In other words, sex selection in order to acquire particular gendered traits demonstrates a misunderstanding of sex and gender that is likely to carry over to situations in which the sex and/or gender of a child does not “align” with societal norms. Again, this is not to claim that such parents are actively transphobic but rather that their decision is guided by a similar logic to that which undermines trans-people by reinforcing the sex-gender binary.

An interesting question arises from these reflections. Should parents be permitted to select intersex children? Intersex people—who represent up to 4 per cent of the population— are not properly characterized as medically male or female (Fausto-Sterling 2008) since (any combination of) their genitals, chromosomes, gonads, or hormones fall outside the confines of the sex binary. Intersex children often do not have any associated medical conditions (in many cases sexual function and even fertility are intact), though all too often intersex bodies are immediately pathologized and subject to “corrective” surgery.

If a child with a particular chromosomal sex is exposed to high levels of particular hormones in utero, the subsequent child may present ambiguous sexual organs. In this way one could contrive an “intersex” child, preserving the child’s fertility and sexual function. Presumably this stands as the ultimate in the sex/gender “variety” that some parents so revere: in principle, with careful calibration, one could produce a series of children, each with a unique genital sex.

Most would not hesitate to condemn a decision to deliberately produce an intersex child. They might cite harm-based arguments akin to those used to critique Sharon Duchesneau and Candy McCullough’s decision to intentionally produce a deaf child (Sandel 2009, 1). Indeed, intersexuality may be limiting in a world whose norms centre on a binary of genital presentation.^6^ Yet choosing a female child in a world in which female bodies are so limited by violence (for example, a third of women globally experience physical or sexual violence at some point (United Nations 2016)) seems troubling too. The latter seems more acceptable only because there are more females than there are intersex or deaf people, so it is not a numerically marginal group, even if females are socially marginal. But that is a matter of degree not kind, and in all three cases the first priority is presumably to limit the harms against these groups, not limit their numbers.

Regardless, it is likely that many objections would note that such an intervention is not natural (even if intersex usually occurs naturally), that children should be accepted whatever the details of their sex, and that it is strange and harmful to be so fixated on the genitals or chromosomes of a child. All of which reflect back on those wishing to select normative sexes for their children.

My point is that sex/gender variety is not what is being sought or celebrated. If it was, the existence (and perhaps production) of children of many different sexes, and associated genders, would be widely tolerated. As it is, parents desiring sex selection for reasons for family balancing are pursuing just two fixed sexes, from which they intend for fixed gender archetypes to stem.

All in all, the literature on sex selection is severely wanting in its understanding of sex and gender and the harms that are inherent to these concepts, particularly with regard to the context of heteronormativity within which they acquire their sense. Once understood, it is clear that those who believe that sex is sufficiently important to form the basis of a decision about what sort of child to raise are acting upon a social difference they erroneously perceive to be intrinsic to the world, which they intend to reproduce, thereby further establishing its (misguided) claim to naturalness.

## A Misuse of Affirmative Action

Affirmative action, also known as “positive discrimination,” involves intervention into the representation of particular human characteristics within a group of interest. This is normally achieved by the imposition of quotas which ensure that people with particular characteristics constitute a particular proportion of the total population. The characteristics in question are normally selected because they are under-represented within that population, therefore quotas artificially over-represent compared with “random” (or, non-quota-driven) occurrences of those characteristics.

Quotas plainly are *not* designed to simply increase diversity along all dimensions, rather, they are generally justified by virtue of their (in principle) capacity to incrementally correct for polarities produced as a result of *structural oppression*. As a rule, quotas are introduced only where *mis*representation is harmful to the group in question. If there is a low occurrence of people with blue eyes within the student population of a university, that would not signal a bias against blue-eyed people. Rather, one would be justified in assuming that the under-representation was random, since blue eyes do not affect university admissions processes and are not likely to have adversely affected people’s lives prior to applying to university. In such cases, quotas would not be justifiable. Equally, if Muslim women were under-represented in very poor populations, it wouldn’t make sense to set a quota to increase their representation, since being poor is not deemed to be in anybody’s best interests. This is in spite of the fact that Muslim women *are* subject to structural oppression more generally.

To summarize: quotas are justifiable in cases in which (a) the under-representation of the characteristic is a result of a structural exclusion and (b) the exclusion of that particular group of people from the population in question constitutes a harm towards those people.

Turning to the question at hand: sex distribution in sibling groups is *random*, not structural, so there is no mandate for the use of quotas (in the absence of additional arguments). Further, one could not argue that it is harmful to males or females as groups that they are not (proportionally) represented within the offspring of a particular family.

Yet it seems that what parents who wish to “balance” their families are advocating could be interpreted to be a form of affirmative action, or, at the very least, has strong echoes of it. Further, it is ironic that were more people to advocate for affirmative action on the basis of gender in its morally rightful places in society, (such as study, employment, and political representation) one might not see quite so many parents feeling the need to select children of particular sexes—it would not *matter* so much in terms of the futures available to that child.

Given that it draws on terminology like “equal,” “fair,” “balance,” “diversity,” “under-representation,” and so on, using “family balancing” as a justification for sex selection of offspring seems to appropriate the terminology, and thereby the logic, of affirmative action, trivializing and undermining its important role in social transformation in particular legitimate contexts. That is, when parents bemoan the lack of males/females amongst their offspring, one is unavoidably reminded of other situations in which males/females are under-represented, and the negative connotations of that under-representation seem to render sex-balance an obviously desirable state. But that is misleading, because the arguments which underwrite our concern at the lack of women in government (say), or men as primary caregivers, simply do not carry over to sex within offspring groups. Discourses and policy around affirmative action and equality have successfully seeped into the public consciousness and have impressed upon us the importance of sex/gender balance, without necessarily carrying over the nuance of justification. Family balancing fallaciously and misleadingly capitalizes on that emphasis, inheriting a presumed acceptability by association. This links back to the note on the rhetorical power of “balance” and “variety” in section three, with “balance” earning a similar moral valence to “equality,” and variety to “diversity.”

Through empirical work, McGowan and Sharp (2013, 289–230) describe how parents seeking family balancing tend to “understand justice in individualist and familial terms rather than in terms of social justice for women and girls or for children who are the product of sex selection.” In other words, those seeking family balancing *do* conceive of their choice as delivering justice. As such, they conceptualize the under-representation of a particular sex within their family as unjust—not to that sex as a whole but to the parent(s) of those children. Even if this is not an intentional misuse of ordinary notions of the injustice of the under-representation of a particular sex, it is certainly a misunderstanding that obfuscates the issue and ignores broader social harms.

## An Abuse of Reproductive Autonomy

Those arguing for sex selection (for “family balancing” or otherwise) sometimes start from the belief that women should have optimal control over their reproductive lives in order to realize the right to bodily autonomy. The reasoning goes that women’s bodies should only ever be used to carry a child they have *chosen* to carry, which might be interpreted as including the right to select the sex of that child.

But arguments from reproductive autonomy fail in this case for precisely the reason they succeed in other cases. Reproductive autonomy is defensible and important because it allows women to recover control of their bodies, which have been under the regulation of patriarchal social values, often via sexist state legislation. As with any right, the right to reproductive autonomy must be demarcated by its impingements on the freedoms of others. An open-ended, unlimited formulation of the “right to choose” is self-undermining. Since the right to choose the sex of children entrenches heteronormativity, which harms women’s rights in so many other ways, there is a substantive tension. And combating gender roles and biological determinism seems to be the more fundamental good.

“Choice” has been prioritized by feminists because there has been a lack of choice in such a way as to undermine women’s agency and preclude their right to control over their own bodies. Yet when choices are won back, they must not be exercised in ways that are harmful to the choices of others, via precisely the ideological mechanism—sexism—that was used to limit choice in the first place.

One particular challenge within this debate is that proponents of choice are generally not informed by feminist values. Rather, within the literature on sex selection, the choice narrative is dominated by libertarian commentators (e.g. Harris 2005; Robertson 2001; Savulescu 1999) whose analyses are not cognizant of, or at the very least do not prioritize, feminist concerns. Rather, these writers work on the assumption that the right to personal choice is the most fundamental concern. What they fail to consider is the way in which some personal choices reinforce cultures in which other personal choices cannot easily be made. That is, allowing parents to choose their children’s sexes fortifies a false and harmful belief about what a particular sexed body can do and be in the world, and can offer within a parent–child relationship. Moreover, since the choice that parents think they are making is founded on a falsehood—that only a child of a particular sex can offer a particular gendered experience—this cannot be claimed to be a genuine enactment of autonomy, characterized as it is by false consciousness (c.f. Browne 2017b). Further, to the extent that their desires (intend to) constrain the child’s development in order to ensure the presence of particular gendered properties, they can be seen to be denying autonomy to others.

Robertson (2001, 6) asks whether “the straighter path to equal rights is to respect female reproductive autonomy whenever it is exercised, even if particular exercises of autonomy are strongly influenced by the sexist norms of her community.” In order to see how indefensible this is, consider whether racism expressed by people of colour would be permissible within an anti-racist movement, on the basis that the voices of people of colour have been silenced. Within society more generally, and medicine specifically, we ought to be concerned with women’s reproductive autonomy, precisely because it has been denied because of sexism. But to permit sexism as an exercise of reproductive autonomy cannot be acceptable. This can be argued even from within a libertarian framework in which freedom in matters of personal choice is deemed to be axiomatic, since sexism limits all our choices and women’s particularly.

Still, it is important to note that resistance to regulation of sex selection often stems from legitimate concerns about the erosion of abortion rights more generally. Some of the most vocal critics of sex selection are anti-choice lobbyists, who have commandeered the widespread criticism of sex-selective abortions (largely due to concerns about son preference) in order to buttress their anti-choice agenda (Mohapatra 2015). This, along with concerns about particular groups of women who may be criminalized if sex-selective abortion is made illegal, has led to some pro-choice interlocutors (such as the British Pregnancy Advisory Service (2015), who released a statement opposing attempts to criminalize sex selective abortions) resisting prohibition of sex-selective abortion, which includes for reasons of “family balancing” (Bhatia 2010; Moazam 2004). Of course, holding an ethical objection to sex selection for family balancing need not translate into a legislative ban on abortions for that reason. Yet one might wonder whether this can be circumvented by targeting screening, rather than abortion; that is, by considering not only whether new technologies ought to be legalized for non-medical purposes but whether older technologies—specifically, those of prenatal sex determination—ought to continue to be legal for non-medical reasons (c.f. Goodkind 1999). Browne (2017a) makes a very convincing case for health professionals refusing to reveal the sex of a foetus, since doing so is a form of misinformation (which entrenches heteronormativity), due to the discrepancy between the *gender* information the parents seek and the *sex* information that is instead provided.

## Conclusion

Sex selection for “family balancing” has three unwelcome consequences. It (a) leads to over-medicalization, (b) entrenches heteronormativity, and (c) appropriates both affirmative action and reproductive autonomy.

Together, these constitute a serious harm against all those whose freedoms are restricted by heteronormativity, all those who are helped by legitimate applications of affirmative action and the struggle for reproductive autonomy, and all those whose well-being is risked by the new ideals, distractions, and dependencies created by over-medicalization.

In short, sex/gender selection is morally problematic largely because *sex/gender* are themselves morally problematic. And reproductive autonomy is valuable *precisely because* it militates against sex-related oppression and has permitted women to reclaim legitimate control over their bodies. For this reason, using the right to reproductive autonomy as a way of defending the right to sex selection is question-begging: it is to use a tool for liberation in order to perpetuate the binary that contributed to the original oppression.

Since sex selection for “family balancing” is generally characterized as the least pernicious motivation for sex selection, its indefensibility weakens the case for sex selection as a whole. On reflection, this should be unsurprising; it would be strange indeed if one found that it became morally acceptable to sex-select a child simply because of the existence of other children and the particularities of their genitals. As it is, whatever the familial context, sex selection for non-medical reasons rests on some very troubling logics and must therefore be resisted. The authority and infrastructure of healthcare should not be used to facilitate practices which play upon misunderstandings and perpetuate injustice.

## Footnotes

1 There is a notable exception to this trend. In the United States, most of those using Microsort sperm sorting request a female embryo. However, the reason for this is likely to be linked to the fact that Microsort promises higher success rates for female embryos: a 91 per cent success rate, compared with a 76 per cent success rate for males (Grady 2007). A 2011 survey by U.S.-based research consultancy Gallup Inc. found that if only permitted to have one child, 40 per cent of participants would prefer a son and 28 per cent a daughter (Newport 2011).

2 Consider that sons will have higher lifetime earnings, have access to a wider range of professional opportunities, will not incur dowry payments (where relevant), will in most cases keep the family name, are unlikely to be subject to sexual and/or domestic violence, etc.

3 The requirements have since changed and, reassuringly, no longer require the couple to be married, but I include McMillan’s endorsement since he presumably agreed with this additional requirement, given that the requirement still applied at the time of the paper’s publication. I do so in order to demonstrate a prejudice towards heteronormativity within the provision of reproductive technologies themselves, and amongst many interlocutors who uncritically endorse them.

4 In institutional settings, chromosomal sex may play a role, but that is a very different discussion, which I take up in section 5.

5 I say “in the first instance” because some of the most influential and robust gendering of small children will be enacted by influences outside the home and will likely happen in spite of parents’ aspirations towards gender-neutral parenting. That is “prospective sex selectors must make their decisions in the real social world, an environment that may well be sexist and which will almost certainly impact on future children’s characters whether the parents like it or not” (Wilkinson 2008, 387). One then might argue the following: I don’t intend to gender my children, but I know that they will likely nonetheless end up gendered in accordance with how their sexed-bodies are construed. I do not want a child who might be violent, thoughtless, and irresponsible, as young men are often socialized to be, and I do not feel that my parenting can offset this powerful norm. I am therefore selecting a female child. This is an important argument, but note that it does nothing to undermine arguments against sex selection for *family balancing* specifically, since parents who want variably gendered children are likely to encourage gendered properties alongside, and in accordance with, external influences.

6 Sparrow (2013) carefully considers the ethics of the inverse situation: that of selecting *against* intersex chromosomes, carefully weighing the virtue of challenging the sex binary by resisting the reduction of the intersex population against the cost of personal suffering (to parents and children) due to the marginalization of non-normative genitals. He makes the important point that the “political project of combating such injustices, seems to demand that parents sacrifice the interests of their children for the sake of the larger social good” (34). I find this point compelling and uncomfortable (as does he) but do not have space to explore it further here.
